# Burden of malaria in pregnancy in Jharkhand State, India

**DOI:** 10.1186/1475-2875-8-210

**Published:** 2009-09-03

**Authors:** Davidson H Hamer, Mrigendra P Singh, Blair J Wylie, Kojo Yeboah-Antwi, Jordan Tuchman, Meghna Desai, Venkatachalam Udhayakumar, Priti Gupta, Mohamad I Brooks, Manmohan M Shukla, Kiran Awasthy, Lora Sabin, William B MacLeod, Aditya P Dash, Neeru Singh

**Affiliations:** 1 Center for Global Health and Development, Boston University School of Public Health, Boston, MA 02118, USA; 2 Department of International Health, Boston University School of Public Health, Boston, MA, USA; 3 Section of Infectious Diseases, Department of Medicine, Boston University School of Medicine, Boston, MA, USA; 4 National Institute for Malaria Research Field Station, Jabalpur, Madhya Pradesh, India; 5 Division of Maternal-Fetal Medicine, Department of Obstetrics and Gynecology, Massachusetts General Hospital, Boston, MA, USA; 6 Center for Leadership and Management, Management Sciences for Health, Cambridge, MA 02139, USA; 7 Malaria Branch, Division of Parasitic Diseases, National Center for Infectious Diseases, Centers for Disease Control and Prevention, USA; 8 National Institute for Malaria Research, Delhi, India; 9 Regional Medical Research Centre for Tribals (Indian Council for Medical Research), Jabalpur, India

## Abstract

**Background:**

Past studies in India included only symptomatic pregnant women and thus may have overestimated the proportion of women with malaria. Given the large population at risk, a cross sectional study was conducted in order to better define the burden of malaria in pregnancy in Jharkhand, a malaria-endemic state in central-east India.

**Methods:**

Cross-sectional surveys at antenatal clinics and delivery units were performed over a 12-month period at two district hospitals in urban and semi-urban areas, and a rural mission hospital. Malaria was diagnosed by Giemsa-stained blood smear and/or rapid diagnostic test using peripheral or placental blood.

**Results:**

2,386 pregnant women were enrolled at the antenatal clinics and 718 at the delivery units. 1.8% (43/2382) of the antenatal clinic cohort had a positive diagnostic test for malaria (53.5% *Plasmodium falciparum*, 37.2% *Plasmodium vivax*, and 9.3% mixed infections). Peripheral parasitaemia was more common in pregnant women attending antenatal clinics in rural sites (adjusted relative risk [aRR] 4.31, 95%CI 1.84-10.11) and in those who were younger than 20 years (aRR 2.68, 95%CI 1.03-6.98). Among delivery unit participants, 1.7% (12/717) had peripheral parasitaemia and 2.4% (17/712) had placental parasitaemia. Women attending delivery units were more likely to be parasitaemic if they were in their first or second pregnancy (aRR 3.17, 95%CI 1.32-7.61), had fever in the last week (aRR 5.34, 95%CI 2.89-9.90), or had rural residence (aRR 3.10, 95%CI 1.66-5.79). Malaria control measures including indoor residual spraying (IRS) and untreated bed nets were common, whereas insecticide-treated bed nets (ITN) and malaria chemoprophylaxis were rarely used.

**Conclusion:**

The prevalence of malaria among pregnant women was relatively low. However, given the large at-risk population in this malaria-endemic region of India, there is a need to enhance ITN availability and use for prevention of malaria in pregnancy, and to improve case management of symptomatic pregnant women.

## Background

Malaria is a disease of global importance that results in 300-660 million cases annually and an estimated 2.2 billion people at risk of infection [1]. Approximately 2.5 million malaria cases are reported annually from South Asia, of which 76% are reported in India [2,3]. Malaria is endemic throughout India with 95% of the population at risk of infection [4]. Infections caused by *Plasmodium falciparum *have increased in India in recent years [2,5].

Malaria in pregnancy (MIP) poses substantial risk to the mother, foetus and neonate. In settings with either stable or unstable transmission, MIP has serious public health consequences. In areas of stable malaria transmission, clinically symptomatic infections are rare and the main consequence is an increased risk of maternal anaemia [6,7] low birth weight (LBW) infants [8] and infant deaths [9]. In areas with low or unstable malaria transmission, pregnant women have little acquired immunity to malaria and are, therefore, at increased risk of symptomatic malaria, severe malaria with central nervous system complications, anaemia, and adverse birth outcomes, such as abortion, preterm labor and stillbirths [10,11].

Past MIP studies in India have demonstrated the important contribution of malaria to maternal and neonatal morbidity and mortality [4,12]. Although preliminary results from earlier studies carried out in central India suggest that both *P. falciparum *and *P. vivax *are associated with adverse pregnancy outcomes, these studies primarily focused on symptomatic pregnant women [12,13]. Relatively little information is available from India about placental malaria, which is associated with an increased risk of neonatal and infant mortality [11,14]. Given the limited information on asymptomatic malaria and placental malaria in India, this study was undertaken in order to better define the burden of MIP, the prevalence of asymptomatic malaria, and the relative contribution of *P. falciparum *and *P. vivax *during pregnancy and at delivery. The study was conducted in the state of Jharkhand in east India, with the ultimate goal of enhancing the development of evidence-based policies to reduce the burden of disease due to MIP in this region of India.

## Methods

### Study site/design

**Figure 1:**
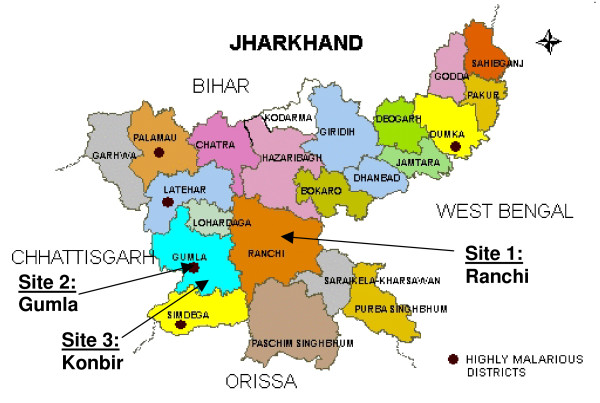
**Map of Jharkhand with study sites**.

This study consisted of a series of cross-sectional surveys conducted in three hospitals (Sadar, Civil and Ursula Mission) in two districts in Jharkhand, India (Figure 1). Jharkhand had a yearly average slide positivity rate (SPR) for symptomatic individuals of 6.8% over the last three years with *P. falciparum *accounting for 44% of the cases [15]. The Jharkhand State Vector Borne Disease Control Programme benefited from the Enhanced Malaria Control Project (EMCP) funded by the World Bank from 1997 to 2005 [16,17]. Most districts, including the two study districts described below, were targeted by this project, which included vector control with larvicides and indoor residual spraying (IRS) with dichloro-diphenyl-trichloroethane (DDT) or synthetic pyrethroids; distribution of insecticide-treated bed nets (ITN); and early case detection and prompt treatment of malaria (any species) with chloroquine and primaquine (chloroquine alone for pregnant women).

The two rural study sites were selected in the district of Gumla (total population as of 2005 was 684,383), which at the time of the study, was considered to be a malaria-endemic area of the state of Jharkhand. In contrast, the third site, Ranchi District (total population as of 2005 was 1,258,306), was selected to represent an urban district with low transmission of malaria. Thus, the three sites were meant to provide a reasonable representation of typical conditions that would be found in Jharkhand. The District Level Household and Facility Survey conducted between December 2007 and April 2008 revealed that 56% of women had at least one antenatal clinic (ANC) visit and 18% overall had institutional deliveries including 59% in urban areas but only 13% in rural settings[18] Sadar Hospital, the district hospital for Ranchi District, serves a predominantly urban population and has a separate obstetric unit with 30 beds, with a high volume of annual deliveries ranging from an average of 2500 to 3000 per year in 2005 to 2008. The Sadar Hospital also has a high volume of ANC visits including an average of 3200 to 3600 per year from 2005 to 2008. Civil Hospital, the district hospital for Gumla District, is a 40-bed hospital serving a semi-urban population, which has 2,000 ANC visits and about 1,000 deliveries annually. The Ursula Mission Hospital, situated in Konbir in Gumla District, serves a predominantly rural population and has 1200 ANC visits and about 300 deliveries each year.

The SPR in Ranchi District was 7.2% in 2005 [15]. IRS in Ranchi is primarily done with DDT. The SPR in Gumla District in 2005 was 3.4% but had been substantially higher in the recent past, ranging from as low as 10% to as high as 19.7% between 1997 and 2003 [15]. The IRS programme in Gumla uses synthetic pyrethroids. Konbir is situated close to the border of Simdega, a highly malarious district with a SPR of 14% in 2005.

### Screening and enrollment

The study had three components with recruitment targeted to women presenting to ANC, delivery units (DU), or the inpatient antepartum ward. For the ANC component, pregnant women aged ≥15 years who reported to the study site for routine care were screened and enrolled. Those who had previously participated in this component of the study or were unwilling to provide written informed consent were excluded. For the DU component, women aged ≥15 years who presented for delivery and were willing to provide written informed consent were enrolled. For inpatients, pregnant women with an admission diagnosis of malaria, anaemia, or a febrile illness of unknown origin were screened for study participation. Those whose malaria-related diagnosis was confirmed or who were treated for malaria were enrolled after obtaining informed consent. Case report forms and study procedures were evaluated in a pilot study and modified accordingly.

### ANC procedures

Trained study personnel interviewed the enrolled women and collected information on socio-demographic characteristics (i.e., date of birth, socio-economic status, literacy); reproductive history including gravidity; history of fever and anti-malarial drug use; and use of anti-malarial prevention measures. A complete physical examination including the determination of gestational age from the height of fundus, measurement of axillary temperature with digital thermometer, and other vital signs was also performed. Blood was obtained by finger-stick for malaria blood film preparation, rapid diagnostic test (RDT), and haemoglobin determination. Women with positive RDT results or who were anemic were referred immediately to the hospital physician for treatment. The clinic staff was informed of additional parasitaemic individuals identified through blood smears so that they could be appropriately treated.

### DU procedures

Pregnant women enrolled at the DUs were interviewed, with data collection focused on socio-demographic characteristics, obstetric complications, history of fever and anti-malarial use during pregnancy, and the use of anti-malarial prevention measures. Blood was obtained by finger-stick soon after delivery for haemoglobin determination, RDT, and malaria blood film preparation. Placental blood by aspiration and an impression smear from the maternal side of the placenta were obtained to determine placental parasitaemia by thick smears and RDT. A drop of cord blood was also taken to prepare a blood smear. All neonates were weighed with an electronic digital scale to the nearest 10 grams and the gestational ages of all live births were estimated within 24 hours of delivery by means of a modified Ballard examination [19]. Women with positive RDT or blood smear results were referred for treatment.

### Inpatient procedures

Enrolled subjects were interviewed and information on socio-economic status, reproductive history including obstetric history, history of fever and anti-malarial drug use, and the use of anti-malarial prevention measures was collected. Data including recorded clinical signs, results of laboratory investigations, treatments administered, admission and discharge diagnosis and outcome of admission were extracted from the subject's hospital record.

### Laboratory procedures

Thick and thin smears prepared from peripheral blood of ANC and DU subjects, placental blood and placental impression from DU subjects, and cord blood were Giemsa-stained and examined under high power. Asexual parasite forms were counted until a minimum of 200 white cells had been counted. Slides were considered negative only if no parasites were seen after identifying 500 leukocytes. Parasite densities were estimated using an assumed total white blood cell count of 8,000 leukocytes/μL of blood [20]. The thin film was used to identify the *Plasmodium *species. All smears were re-checked by a member of the parasitology laboratory at the National Institute of Malaria Research Field Station in Jabalpur. The First Response Malaria Pf/Pv test (PMC, Mumbai, India), a parasite lactic dehydrogenase antigen test, was used to perform the RDTs. This test has a sensitivity of 93% and specificity of 85% for detection of malaria in non-pregnant individuals [21]. The First Response Malaria Pf/Pv test has also been evaluated as a screening tool in pregnant women. Compared to expert microscopy, this test had a sensitivity and specificity of 95% and 99.6% for *P. falciparum *and 69% and 99.4% for *P. vivax*, respectively [22]. A portable HemoCue machine (Ängelholm, Sweden), using a control with each assay, was used for haemoglobin determinations.

### Study definitions

The following definitions were used:

Peripheral parasitaemia: presence of asexual *P. falciparum *or *P. vivax *parasitic forms on blood smears or positive RDT.

Placental malaria: presence of malaria parasites on impression smear of maternal side of placenta or by RDT.

Symptomatic malaria infection: history of fever within the last week or temperature ≥ 37.5°C associated with the presence of asexual forms of *P. falciparum *or *P. vivax *on thick blood smear or a positive RDT.

Severe malaria: a malaria attack associated with any of the following: cerebral malaria, severe anaemia, renal failure, pulmonary oedema, hypoglycaemia, shock, spontaneous bleeding, or repeated convulsions[23]

Anaemia: haemoglobin <110 g/L.

Severe anaemia: haemoglobin <70 g/L.

Low birth weight: birth weight < 2,500 g.

Prematurity: gestational age <37 weeks as assessed by Ballard examination [19].

Stillbirth: death of foetus prior to delivery.

### Ethical clearance

The study was approved by the Institutional Review Boards of Boston University and the Centers for Disease Control and Prevention, the Ethics Committee of the National Institute of Malaria Research (NIMR) in India, the Scientific Advisory Committee of the NIMR and the Health Ministry Screening Committee of the Indian Council of Medical Research.

### Data management and analysis

All case report forms were checked for completeness and inappropriate or illogical responses. The forms were double-entered using CS-Pro, with range, consistency, and edit checks built into the data entry programme for quality control. The two databases were validated and all inconsistencies and differences were resolved. Statistical analyses were performed using SAS software version 9.1 (Cary, North Carolina). Categorical data are presented as frequency counts (percent) and compared using the chi-square or Fisher's exact statistic as appropriate. Continuous data are presented as means (± standard deviation) and compared using the t-test or analysis of variance as appropriate. Since most participants did not know their exact date of birth, we have presented participants' ages in ranges based on their estimations. Risk factors for either *P. falciparum *or *P. vivax *parasitaemia were evaluated by univariate analysis and then adjusted for significant predictors in multivariate analysis.

## Results

Recruitment and enrollment took place from December 2006 to December 2007. Of 2499 pregnant women screened during their ANC visits, 60 were not eligible because they had previously participated in the study, 53 refused to provide informed consent, and 2,386 were enrolled. In the DU, 739 pregnant women were screened and all of them were eligible, although 21 refused to provide consent and therefore 718 were enrolled.

### Antenatal clinics

Most pregnant women attending ANC were in the 20 to 34 year old age range and had some level of formal education (Table 1). The vast majority of participants were Hindi speaking (99.4%) and non-smoking (99.9%). Most owned their own home (73.4%) and were engaged in household work (76.7%) with a small proportion involved in farming (10.7%). They had attended a median of one ANC visit (range 0-9) during their current pregnancy and slightly more than a third were primigravidae. Slightly more than half of participants presented to the ANC in the latter half of pregnancy whereas 46.3% presented prior to 20 weeks. Less than half of the participants reported taking iron/folate supplements (39.6%) while 27.3% were taking multivitamins. In terms of malaria prevention activities, most pregnant women reported having untreated bed nets in their homes, and using them recently, but very few had ITNs (Table 2). Similarly, only 14 of the women were taking prophylaxis for malaria and most of them (10/14, 71%) were unable to identify the drug they were taking. Of those who were able to identify the drug they were using, 75% (3/4) were taking chloroquine.

**Table 1:** Baseline characteristics of pregnant women attending antenatal clinics and delivery units

***Characteristic***	***Antenatal clinics*** ***n *= 2386**	***Delivery units*** ***n *= 718**
	**n^† ^(%)**	**n^† ^(%)**
Age (years)		
<20	305 (12.8%)	67 (9.3%)
20-34	1,978 (82.9%)	614 (85.5%)
≥35	103 (4.3%)	37 (5.2%)
Prior pregnancies		
Primigravid	875 (36.7%)	306 (42.6%)
Secundigravid	628 (26.3%)	174 (24.2%)
Multigravid*	882 (37.0%)	238 (33.2%)
Gestational age at enrollment (weeks)**		
< 20 weeks	1,104 (46.3%)	n/a
20-36 weeks	1,239 (51.9%)	42 (7.1%)
≥ 37 weeks	43 (1.8%)	637 (92.9%)
Caste		
Schedule caste	234 (9.8%)	74 (10.3%)
General caste	678 (28.4%)	189 (26.3%)
Other backward caste	683 (28.7%)	192 (26.8%)
Scheduled tribal	789 (33.1%)	263 (36.6%)
Education		
No formal schooling	621 (26.0%)	217 (30.2%)
Attended school any length of time	1,764 (74.0%)	501 (69.8%)
Socioeconomic characteristics		
Owns TV	1,196 (50.1%)	293 (40.8%)
Owns bicycle	1,635 (68.5%)	512 (71.3%)
Owns house	1,751 (73.4%)	585 (81.5%)
Owns refrigerator	104 (4.4%)	16 (2.2%)
Roof material		
Mud	1,317 (55.2%)	482 (67.1%)
Corrugated iron/asbestos sheet	699 (29.3%)	149 (20.8%)
Cement/concrete	350 (14.7%)	82 (11.4%)
Other	20 (0.8%)	5 (0.7%)
Wall material		
Mud/sand/dung	1,193 (50.0%)	452 (63.0%)
Mud bricks	131 (5.5%)	33 (4.6%)
Cement bricks	1,008 (42.2%)	212 (29.5%)
Other	54 (2.3%)	21 (2.9%)
Primary cooking fuel		
Wood	1,428 (59.9%)	502 (69.9%)
Charcoal	441 (18.5%)	107 (14.9%)
Gas	480 (20.1%)	95 (13.2%)
Other	37 (1.5%)	14 (2.0%)

^† ^Numbers may not add to sample size secondary to missing data.

* Defined as 3 or more pregnancies

** For ANC enrollees, gestational age assessed by fundal height. For DU enrollees, gestational age was assessed by Ballard score. Infants that were stillborn did not have a Ballard examination performed.

**Table 2:** Use of malaria prevention measures by pregnant women attending antenatal clinics and delivery units

***Prevention measures utilized***	***Antenatal clinics*** ***n *= 2386** ***n, (%)***	***Delivery units*** ***n *= 718** ***n, (%)***
Bed net in household	2,055 (86.2%)	597 (83.3%)
Insecticide-treated bed net in household	79 (3.3%)	22 (3.1%)
Sleeps under bed net most nights	1,702 (82.8%)	439 (73.7%)
Taken malaria prophylaxis in pregnancy	14 (0.6%)	2 (0.3%)
Indoor residual spraying of home with insecticide	1270 (53.5%)	418 (58.5%)

A positive diagnostic test for malaria was obtained in 1.8% (43/2382) of the total cohort (Table 3). Peripheral smears were not performed for four pregnant women. Blood smears for malaria were positive in 1.3% of pregnant women while an additional 11 (0.5%) women had positive RDTs. The mean density of parasitaemia in the 32 women with positive blood smears was 57,145 asexual forms/μL (range 200-376,000). *P. falciparum *was identified in 53.5% of parasitemic individuals while *P. vivax *was found in 37.2% and 9.3% of infections were mixed. Peripheral parasitaemia was over three times more likely among women living in rural areas when compared with those from urban or semi-urban sites (OR 3.45, 95% CI 1.88-6.32), and among primigravidae and secundigravidae relative to multigravidae (OR 3.69, 95%CI 1.55-8.77). Primigravidae had higher parasite densities than women with one or more past pregnancies but this difference was not statistically significant (mean ± SD of 70,034 ± 107,625 vs. 16,090 ± 66,883 asexual forms/μL, respectively, p = 0.47). Parasitaemia was more commonly encountered in pregnant women who had a history of fever within the week prior to enrollment or were febrile at the time of the study visit (5.5% vs. 1.1%, p < 0.001). Overall 51.2% (22/43) of the pregnant women with a positive malaria diagnostic test at the time of ANC visit were symptomatic. The majority of positive malaria tests occurred from April to December with the greatest number in July, corresponding to the monsoon season.

**Table 3:** Parasitaemia, reported fever, and anaemia among pregnant women attending antenatal clinics and delivery units

	***Antenatal Clinics***	***Delivery Units***
	**n (%)**	**n (%)**
Peripheral parasitaemia^¶^		
Overall	43/2,382 (1.8%)	12/717 (1.7%)
Falciparum	23/2,382 (1.0%)	9/717 (1.3%)
Vivax	16/2,382 (0.7%)	2/717 (0.3%)
Mixed	4/2,382 (0.2%)	1/717 (0.14%)
By site		
Urban (Ranchi)	7/935 (0.8%)	2/254 (0.8%)
Semiurban (Gumla)	15/907 (1.6%)	4/183 (2.2%)
Rural (Konubir)	21/525 (4.0%)	6/280 (2.1%)
By gravidity	19/875 (2.2%)	6/306 (2.0%)
Primigravid	18/628 (2.9%)	5/173 (2.9%)
Secundigravid	6/882 (0.7%)	1/238 (0.4%)
Multigravid**		
Placental parasitaemia		
Overall	n/a	17/712 (2.4%)
Falciparum		12/712 (1.7%)
Vivax		2/712 (0.3%)
Mixed		3/712 (0.4%)
By site	n/a	
Urban (Ranchi)		2/252 (0.8%)
Semiurban (Gumla)		5/181 (2.8%)
Rural (Konubir)		10/279 (3.6%)
By gravidity	n/a	
Primigravid		9/303 (3.0%)
Secundigravid		5/172 (2.9%)
Multigravid		3/237 (1.3%)
Cord blood parasitaemia	n/a	6/562 (1.1%)
Report of fever within 1 week	400/2,384 (16.8%)	78/715 (10.9%)
Anaemia	1,722/2,378 (72.4%)	424/711 (59.6%)
Severe anaemia	92/2,378 (3.9%)	32/711 (4.5%)

^¶^Parasitaemia defined by presence of parasites on blood or tissue impression smear or by positive RDT.

*ANC: Peripheral blood smears not obtained for 4 women. DU: peripheral blood smears not obtained for 1 woman. Placental blood not obtained for 6 women.

*Defined as 3 or more pregnancies

NA = not applicable

Anaemia was common among ANC participants whereas severe anaemia was rare (Table 3). Anaemia was not associated with malaria (p = 0.77); however, severe anaemia was more common among women with parasitaemia (p = 0.023).

Multivariate analysis was performed in order to identify the association between specific demographic, socioeconomic, and malaria prevention activities and the risk of parasitaemia. Among pregnant women attending ANCs, first/second pregnancies, fever in the past week, and residence in rural areas were significantly associated with peripheral parasitaemia (Table 4).

**Table 4:** Univariate and multivariate analysis of predictors of peripheral parasitaemia among pregnant women attending antenatal clinics

	**Univariate Analysis**		**Multivariate Analysis**
	
	**Peripheral Parasitaemia** **% (Positive/Total)**	**Relative Risk (95% CI)**	**Adjusted^¶^** **Relative Risk (95% CI)**
First/second pregnancies	2.5% (37/1503)	3.62 (1.53 - 8.54)	3.17 (1.32-7.61)
Third or greater pregnancy	0.7% (6/882)	1	
Age < 20	2.0% (6/305)	1.11 (0.47 - 2.60)	
Age ≥ 20	1.8% (37/2081)	1	
Fever within past week	5.5% (22/401)	5.19 (2.88 - 9.34)	5.34(2.89-9.90)
No fever within past week	1.1% (21/1985)	1	
Bednet use*	1.8% (31/1702)	1.29 (0.50 - 3.29)	
No bednet use	1.4% (5/354)	1	
Indoor residual spraying	1.8% (23/1270)	1.00 (0.55 - 1.81)	
No indoor residual spraying	1.8% (20/1104)	1	
Rural	4.0% (21/527)	3.37 (1.87 - 6.07)	3.10(1.66-5.79)
Not rural	1.2% (22/1859)	1	
Tribal caste	2.8% (22/789)	2.12 (1.17 - 3.83)	1.67 (0.90-3.11)
Not tribal caste	1.3% (21/1595)	1	
No formal education	1.3% (8/621)	0.65 (0.30 - 1.39)	
Formal education	2.0% (35/1764)	1	
Homeowner	1.9% (34/1751)	1.37 (0.66 - 2.84)	
Not homeowner	1.4% (9/635)	1	
Mud walls	2.3% (30/1324)	1.85 (0.97 - 3.53)	
No mud walls	1.2% (13/1062)	1	

^¶^Risk ratio adjusted for first/second pregnancies, fever within past week, rural locale, and tribal caste.

* ITN use was not evaluated in this model since these were very rarely used.

### Delivery units

Like the ANC cohort, most pregnant women attending DUs were aged 20-34 years and had some level of formal education (Table 1). All were non-smokers (100%) and nearly all spoke Hindi (99.4%). Most owned their own home (81.5%) and were involved in household work (76.6%); a minority engaged in farming (11.1%). Study participants had attended a median of two ANC visits (range 0-9) and about two-thirds were primigravidae and secundigravidae (Table 1). The majority of pregnant women reported having untreated bed nets in their homes and using them recently but ITN ownership was rare (Table 2). Only two women were taking chemoprophylaxis for malaria and neither knew the name of the medication that they were taking.

Only 1.7% of the women enrolled at the DUs had peripheral parasitaemia (either a positive blood smear and/or RDT).*P. falciparum *was identified in 75% (9/12), *P. vivax *in 17% (2/12), and mixed infection in 8% (1/12). The mean density of parasitaemia in the women with positive blood smears was 14,272 asexual forms/μL (range 280-42,000). The peripheral parasitaemia density was significantly higher for primigravid women than in those who had one or more prior pregnancies (mean ± SD of 22,800 ± 10,866 vs. 5,744 ± 5847 asexual forms/μL, respectively; p = 0.02). A greater proportion of the women presenting to the semi-urban and rural sites were parasitaemic but this difference was not significant (OR 2.78, 95%CI 0.61-12.8) (Table 3). Primigravidae and secundigravidae also were more likely to be parasitaemic, but this difference was not significant (OR 5.57, 95% CI 0.72-43.4). Multivariate analysis revealed a significant association between placental or peripheral parasitaemia and age less than 20 years and fever within the last week (Table 5).

**Table 5:** Univariate and multivariate analysis of predictors of peripheral parasitaemia among women attending delivery units

	**Univariate Analysis**		**Multivariate Analysis**
	**Peripheral Parasitaemia** **% (Positive/Total)**	**Relative Risk (95% CI)**	**Adjusted^¶^** **Relative Risk (95% CI)**
First/second pregnancies	3.8% (18/475)	2.99 (0.89 - 10.06)	
Third or greater pregnancy	1.3% (3/237)	1	
Age <20	7.6% (5/66)	3.06 (1.16 - 8.08)	2.68 (1.03 - 6.98)
Age ≥ 20	2.5% (16/646)	1	
Fever within past week	9.6% (8/83)	4.64 (1.98 - 10.86)	4.31 (1.84 - 10.11)
No fever within past week	2.1% (13/626)	1	
Bednet use*	3.0% (13/437)	1.54 (0.44 - 5.32)	
No bednet use	1.9% (3/155)	1	
Indoor residual spraying	3.9% (16/415)	2.26 (0.84 - 6.10)	
No indoor residual spraying	1.7% (5/293)	1	
Rural	3.9% (11/279)	1.71 (0.73 - 3.97)	
Not rural	2.3% (10/433)	1	
Tribal caste	3.1% (8/260)	1.07 (0.45 - 2.55)	
Not tribal caste	2.9% (13/452)	1	
No formal education	2.8% (6/217)	0.91 (0.36 - 2.32)	
Formal education	3.0% (15/495)	1	
Homeowner	3.5% (20/579)	4.59 (0.62 - 33.93)	
Not homeowner	0.8% (1/133)	1	
Mud walls	3.8% (18/480)	2.90 (0.86 - 9.75)	
No mud walls	1.3% (3/232)	1	

^†^Risk ratio adjusted for fever within past week and age less than 20.

* ITN use was not evaluated in this model since these were very rarely used.

Placental impression yielded one more case (10/712, 1.4%) than placental blood smears (9/712, 1.3%). The mean density of placental parasitaemia in the 10 women with positive smears was 13,200 asexual forms/μL (range 240- 42,000). An additional seven cases of placental parasitaemia were identified by RDT yielding an overall prevalence of placental parasitaemia of 2.4% with non-significantly greater proportions seen at the non-urban sites and in primigravidae and secundigravidae. Symptomatic malaria infections were present in 42% of women with peripheral parasitaemia (5/12) and 41% of those with placental parasitaemia (7/17). Pregnant women with peripheral parasitaemia were more likely to have either a self-reported fever or fever measured at enrollment than those who were aparasitaemic (41.7% vs 11.3%, p = 0.008). Similarly, more women with placental parasitaemia had fever (reported or measured) compared with women whose placentas did not harbour parasites (41.2% vs. 11.0% p = 0.002). As observed in the ANC participants, most episodes of parasitaemia occurred in July and August during the monsoon season.

More than half of the DU participants were anemic but only 4.5% had severe anaemia (Table 3). For DU participants with peripheral parasitaemia, 100% had anaemia as compared to 58.9% of those who did not have parasitaemia (p = 0.002). More women with peripheral parasitaemia had severe anaemia (8.3%) than those without parasitaemia (4.4%) but the difference was not significant (p = 0.43).

Excluding the 6 sets of twins delivered, 20.9% (141/675) of the babies delivered by the DU participants had LBW. There were slightly more LBW infants among mothers with placental malaria, but this difference was not significant (p = 0.53) (Table 6). The overall prevalence of preterm delivery by Ballard score in the DU participants was 5.8% (39/674) and was non-significantly (p = 0.22) higher in the women with placental parasitaemia. Among the participants with placental malaria, the prevalence of stillbirth was more than twice as frequent in those without placental malaria but due to the low rates of infection, this difference was not significant (p = 0.16).

**Table 6:** Prevalence of adverse birth outcomes by presence versus absence of placental parasitaemia among pregnant women enrolled

	***Placental Parasitaemia***	
	**Present** **n^† ^(%)**	**Absent** **n^† ^(%)**
Low birth weight	4/15 (26.7%)	137/657 (20.9%)
Preterm birth	2/15 (13.3%)	37/656 (5.6%)
Stillbirth	2/17 (11.8%)	28/686 (4.1%)
Gestational hypertension	4/17 (23.5%)	195/695 (28.1%)
	Median (range)	Median (range)
Birth weight	2,500 (2,100-3,900)	2,700 (1,300-4,500)
Gestational age	38 (36-40)	38 (34-42)

^†^Denominators vary because the neonate had to be born alive to be weighed and receive a Ballard score. Additionally, twin gestations and women whose placentas were not examined for parasitaemia were excluded from analysis of birth outcomes.

* Gestational hypertension defined as systolic blood pressure > 140 mmHg or diastolic blood pressure > 90 mmHg.

### In-patients

Twenty-seven pregnant women who were admitted and treated for malaria according to national guidelines in the three hospitals were enrolled. They represented 19.2% of pregnant women admitted for any medical treatment other than deliveries during the twelve-month study period. The majority (82%) of the inpatients were between 20 and 34 years old (82%). Six (22%) were primigravidae, 10 (37%) secundigravidae, and 11 (41%) multigravidae. The majority was from scheduled tribes (59%) with the others belonging to general caste (19%), scheduled caste (11%) and other backward castes (11%). Similar to the ANC and DU participants, 78% reported having a bed net in the household and 63% reported sleeping under it most nights although only 7.4% were insecticide-treated. Sixty-seven percent of their homes had been sprayed with insecticide and only one woman had been taking chemoprophylaxis during pregnancy.

Malaria was confirmed by microscopy or RDT in 92.6% (25/27) of the inpatients. *P. falciparum *was the cause of malaria for 84% (21/25) of the pregnant women, while two (8%) had *P. vivax *and two (8%) had mixed infection with *P. falciparum *and *P. vivax*. Eight (29.6%) of the admitted pregnant women had severe malaria (severe anaemia, cerebral malaria) based on WHO criteria [23]. Of the 20 patients whose haemoglobin results were recorded in the medical records, 12 had anaemia, five had severe anaemia, and three were not anaemic. *P. falciparum *was responsible for severe malaria in seven of eight women, while the last had a mixed infection. All admitted pregnant women were treated with parenteral or oral quinine, arteether or artesunate with excellent responses; none died.

## Discussion

There was a relatively low prevalence of malaria among pregnant women attending ANCs and delivering in the study site hospitals in Jharkhand. Previous studies of MIP in India found similar to higher prevalence rates, ranging from 1.4% to 20% [12,24,25] However, these studies focused on pregnant women who were febrile or had a recent history of fever and thus may have had a selection bias towards higher malaria rates. This approach, targeting malaria diagnostic and treatment for symptomatic pregnant women, is consistent with India's National Vector Borne Disease Control Programme guidelines [5]. In contrast, all pregnant women were evaluated in the current study, including those who were asymptomatic. The low prevalence of malaria, especially in the urban and semi-urban study sites, suggests that these areas have low rates of malaria transmission and, therefore, there is a potential risk of outbreaks. Malaria was responsible for about one-fifth of all hospitalizations of pregnant women at the three study sites, suggesting that malaria, especially when caused by *P. falciparum*, is responsible for a substantial portion of serious illness requiring hospital admission for pregnant women in this region.

Malaria occurred more commonly in women in rural areas and those who were in their first or second pregnancy, as has been seen in studies of MIP in sub-Saharan Africa [26]. The higher prevalence at the rural sites may be due to higher transmission, less availability of preventive measures such as ITNs and IRS, and limited access to anti-malarial drugs. Pregnant women in urban areas may have better access to prophylactic or therapeutic anti-malarial drugs through private practitioners and other community sources. However, the majority of study participants had not taken an anti-malarial for treatment during the past week or at any time during their pregnancy.

Overall, there was a substantial burden of anaemia among pregnant women. While the proportion of pregnant women suffering from severe anaemia was relatively small (~4% in the ANC cohort), there was a significant association with malaria. Among women in the DU cohort, there was no association between parasitaemia and severe anaemia. The lack of an association with severe anaemia can be potentially attributed to the smaller sample size in the DU study cohort. Although none of the associations was significant, adverse maternal and birth outcomes including LBW, prematurity, stillbirth, and gestational hypertension all occurred more commonly in pregnant women with parasitaemia. Despite the low frequency of MIP, the large population of Jharkhand, nearly 22 million people [27], means that there are nearly 100,000 women at risk for malaria-associated complications based on the 1.8% prevalence we observed in the ANC population and assuming that 25% of the population are women of child-bearing age. Consistent with this hypothesis, a recent re-evaluation of the worldwide burden of malaria in pregnant women suggested a much higher burden of disease in the Asia-Pacific region than previous estimates [28].

The EMCP was active in both Ranchi and Gumla Districts until 2005 when the program ended. The low prevalence of malaria among pregnant women in the current study might have resulted from enhanced detection and treatment of symptomatic individuals in the community through personnel trained by this program. However, the very low rate of ownership of ITNs suggests that this component of the EMCP has not effectively reached this vulnerable population although it was encouraging to find that many households had bed nets and that they were used on a regular basis. The enhanced provision of ITNs and their regular retreatment are cornerstones of the EMCP [5,16]. Their notable absence among the large cohort of pregnant women in this study, despite both study districts having recently participated in this program and the EMCP guidelines which prioritized ITN delivery to pregnant women and children, suggests that approaches for ITN distribution and enhancing community awareness about the importance of their use need to be addressed. Given the challenges of re-impregnating bednets, the use of long-lasting ITNs would be preferable.

Despite its existence as an official guideline at the time, chloroquine was almost never used for prevention of MIP. Although chloroquine resistance has been rising in India [2,5], this drug was recommended for malaria prophylaxis in pregnant women in high risk areas at the time of the study [29]. This recommendation has since been discontinued. An alternative approach that is commonly used in Africa is intermittent preventive treatment of pregnant women (IPTp) with sulphadoxine-pyrimethamine [30]. Nonetheless, since the intensity of transmission and the prevalence of malaria in pregnant women in Jharkhand are lower than in many areas in sub-Saharan Africa, there does not appear to be an urgent need to implement the use of IPTp. A top priority in India, as a first step, should be improved availability and use of ITNs by pregnant women. If this step alone proves inadequate, then an alternative strategy to IPTp that might be appropriate is the use of intermittent screening and treatment in pregnant women. In this approach pregnant women are screened for malaria parasitaemia at each antenatal visit with either RDTs or blood smears. Treatment for malaria is then provided only if the test is positive. This strategy could potentially reduce the burden of MIP while limiting the potential for anti-malarial resistance to develop due to the widespread use of drugs for chemoprophylaxis. This strategy would be especially effective in urban areas of Jharkhand as 85% of pregnant women have at least one ANC visit and 65% have three or more visits [18]. In contrast, this approach would be less useful in rural areas of the state because only 53% of pregnant women have at least one ANC visit and only 27% have three or more.

One major limitation of this study is that the cross-sectional surveys were facility-based. Since a great part of the malaria burden is thought to occur in marginalized, remote tribal populations, the findings in this study may not be generalizable to all areas and populations within the state of Jharkhand. In addition, because this was a cross-sectional rather than a longitudinal study, the actual burden of MIP in this region may have been underestimated. Thus, the study design did not allow for an understanding of the natural history of parasitaemia in pregnant women or the frequency of malaria attacks during the course of gestation. The use of placental smear in this study instead of histopathology might have underestimated the burden of malaria since histopathology is more sensitive in identifying placental malaria [20,21]. Finally, there is limited information on the use of RDTs for diagnosing placental malaria [31,32]. Since these tests detect antigen, it is possible that they may be detecting malaria antigen in the placenta from chronic or past infections and thus may not conclusively demonstrate the presence of acute placental malaria.

## Conclusion

Given the large at-risk population in this malaria-endemic region of India, there is a need to enhance ITN use for the prevention of MIP. Fortunately, there is already a culture of bed net use. If ITNs become more widely available, they would likely be acceptable to pregnant women barring any problems with their perception of risk associated with the chemicals used to treat the nets. Since the retreatment of ITNs presents many logistical challenges, future efforts should focus on the provision of long-lasting ITNs. An additional challenge will be to increase their availability and to make them affordable for Indians living below the poverty line. There should be a focus on improving case management of symptomatic pregnant women, and evaluating the efficacy and effectiveness of the intermittent screening and treatment strategy. IPTp does not appear to be a rational choice for reducing the burden of MIP, and chloroquine prophylaxis may not be effective due to rising resistance and, in any case, is not being implemented in Jharkhand. This requires enhancing the availability and use of diagnostic tests including RDTs and ensuring the availability of safe and effective drugs for the treatment of pregnant women with malaria.

## Competing interests

Davidson Hamer has one competing interest to declare, specifically equity ownership in Inverness Medical Innovations, a company that produces a malaria rapid diagnostic test. All other authors declare that they have no competing interests.

## Authors' contributions

DHH, BJW, KYA, MD, VU, WBM, and NS contributed to the conception and design of the study. MPH, JT, PG, MIB, MS, KA, APD, and NS all contributed to study implementation and data collection. BJW, MPS, and WBM performed data analyses and DHH, KYA, MD, VU, MIB, JT, and NS assisted with interpretation of data. DHH, BJW, KYA, and NS drafted the manuscript. All authors contributed to and approved the final manuscript.

## Financial support

The United States Agency for International Development (USAID)/India mission provided funding for this study to the Child and Family Applied Research project at Boston University, Boston, by means of the USAID cooperative agreement (GHS-A-00-03-00020-00). The opinions expressed herein are those of the authors and do not necessarily reflect the views of USAID.
